# In vitro and in vivo antioxidant and antihyperglycemic potentials of phenolic fractions of *Syzygium zeylanicum* (L.) DC trunk‐bark

**DOI:** 10.1002/fsn3.3373

**Published:** 2023-04-26

**Authors:** Minh‐Trung Nguyen, Bich Huyen Bui Thi, Shila Maskey, Minh‐Dinh Tran, Quang‐Vinh Nguyen

**Affiliations:** ^1^ Institute of Biotechnology and Environment Tay Nguyen University Buon Ma Thuot Vietnam; ^2^ Faculty of Natural Science and Technology Tay Nguyen University Buon Ma Thuot Vietnam; ^3^ Patan Multiple Campus Tribhuvan University Patan Nepal

**Keywords:** antidiabetic activities, antioxidant activities, phenolic compounds, principal component analysis, *Syzygium zeylanicum* L. (DC)

## Abstract

*Syzygium zeylanicum* L. (DC) (*SZL*) has been used in antidiabetes treatment for ages. However, the scientific evidence of active agents that have antidiabetic activity and response against biological activities is limited. In this study, the active components of SZL trunk‐bark extract (*SZL* extract) were identified using principal component analysis (PCA), and their antidiabetic activities were assessed. The results indicated that the ethyl acetate fraction (EAF) had the highest concentration of phenolic compounds, antioxidants, and antihyperglycemic activities in the postprandial zebrafish model. The major antioxidant contributors were gallic acid, catechin, epicatechin, ellagic acid, quercetin, caffeine, and apigenin, and their concentrated levels reduced α‐amylase inhibitory activity, whereas rutin and ethyl gallate influenced the α‐glucosidase inhibitory activity. This study showed the bio‐functional properties of active phenolic compounds present in the *SZL* extract, potentially serving as a functional food to control hyperglycemia.

## Graphical abstract

This study aims to investigate the primary bioactive component of *Syzygium zeylanicum* (L.) DC trunk‐bark during extraction and fractionation and assess their in vitro antioxidant and antidiabetes activity, the phenolic chemical profile, and their influence on the bioactivity of the extract and its fraction. Furthermore, the antihyperglycemic activity was evaluated on zebrafish model. The results showed the bio‐functional properties of active phenolic compounds present in the SZL extract and potentially serve as a functional food to control hyperglycemia.

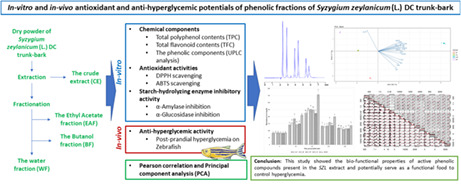

## 1 INTRODUCTION

According to International Diabetes Federation reports, the global prevalence of diabetes mellitus (DM) reached 9.5% (463 million adults) in 2019 to 10.5% (536.6 million adults) in 2021, rising to 12.2% (783.2 million) in 2045 (Sun et al., 2022), despite global efforts to control the disease. A multi‐therapeutic approach to medication is regarded as the most proper approach for DM prevention and treatment (Tiwari, 2015). Several pieces of evidence from traditional knowledge and recent scientific studies show that medicinal plant extract is the most appropriate therapeutic approach with a multi‐impact mechanism with safety (Bindu & Narendhirakannan, 2019; Willcox et al., 2021). Of these, controlling postprandial hyperglycemia via the modulation of starch‐hydrolyzing enzymes, using α‐amylase and α‐glucosidase inhibitors derived from a medicinal plant, is an effective prophylactic therapy for type 2 DM (Bashary et al., 2020; Dirir et al., 2021). Besides, the roles of oxidative stress and inflammation in the development and progression of DM and its consequences were highlighted in a recent study (Kanwugu et al., 2022). Thus, the plant extracts contain α‐amylase and α‐glucosidase inhibitors, as well as natural antioxidants, which provide effective therapy for diabetes treatment by influencing human physiological function (Shori, 2015).

*Syzygium zeylanicum* (L.) DC. (*SZL*) is a valuable medicinal plant found in secondary populations across Vietnam, from Kon Tum to the South. According to recent reports, *SZL* has the potential to be used in diabetic treatment due to its ability to resist oxidation and inhibit starch hydrolyzing enzymes. *SZL* leaf extract was discovered as a novel promising material that has proven outstanding performance in terms of α‐glucosidase inhibition and antioxidant activities (Mai et al., 2007). Our preliminary findings showed that the methanolic extract of *SZL* trunk‐bark had greater α‐glucosidase inhibitory activity than acarbose and effectively decreased blood glucose levels in streptozotocin‐induced diabetic rat models (Nguyen et al., 2019). However, the active components and their characterization are still unknown. Moreover, several recent investigations discovered that the fractionation approach profoundly influenced the bioactive component contents and their related biological activities (Franco et al., 2020; Nguyen Quang et al., 2021; Yadav et al., 2011). However, the influence of various solvent fractionation on the bioactive substances from *SZL* and their related bioactivities has not yet been investigated. Therefore, this study aimed to evaluate the antioxidant and antidiabetic activities of the fractionated extracts of *SZL*. In addition, the correlation between the phenolic compounds as the main components present in the fractions of the extract and their biological activities was identified by PCA.

## 2 MATERIALS AND METHODS

### 2.1 Chemicals

Folin–Ciocalteu, gallic acid, catechin, epicatechin, caffeine, ethyl gallate, rutin, ellagic acid, quercetin, quercitrin, apigenin, chlorogenic acid, DPPH, ABTS, acarbose, pancreatic α‐amylase, and yeast α‐glucosidase were purchased from Sigma‐Aldrich. Maltodextrin (MAX1000® series, Matsutani Chemical Industry Co., Ltd.) was acquired from DKSH Management Ltd., Vietnam. Xilong Scientific Co. Ltd. supplied ethanol, methanol, and acetic acid. All chemicals and reagents were of analytical grade.

### 2.2 Plant sample

The trunk‐bark of SZL collected from Easo Nature Reserve, Ea Kar District, Dak Lak Province, Vietnam was dried at room temperature in a well‐ventilated environment, powdered, sealed in PE bags, and stored at −30°C until further use.

### 2.3 Preparation of crude extract and fractions

The crude extract (CE) was prepared by immersion method as reported by Nguyen, Van Chuyen, et al. (2022), with slight modifications for using ultrasound‐assisted extraction. In brief, the powdered trunk‐bark of *SZL* was steeped in 50% aqueous ethanol solution at a ratio of 1:10 w:v for 2 min before being sonicated for 15 min at 28 kHz using a micro‐tipped probe 10 mm in diameter (Vietsonic, VS28H). The mixture was entirely extracted for 24 h at room temperature before being filtered using filter paper (No.1). The residues were extracted a second time using the same procedure. The extracts were then combined and concentrated in a rotary evaporator (IKA) at 60°C until half of the volume was recorded. The extract solution was further fractionated with different polar solvents using the procedure described by Nguyen Quang et al. (2021). The crude extract (CE), n‐hexane (HF), chloroform (CF), ethyl acetate (EAF), n‐butanol (BF), and water fractions (WF) were fully dried by an Operon freeze dryer (Operon FBD‐5503, Korea) at – 56°C and 0.001 Mbar pressure and stored at – 30°C until further use.

### 2.4 Determination of the total polyphenol and flavonoid contents

The total polyphenol and flavonoid contents (TPC and TFC) were estimated using methods described in a previous publication (Nguyen et al., 2016).

### 2.5 Determination of antioxidant capacity

The capacity of DPPH^•^ and ABTS^•+^ radical scavenging was evaluated using the approach outlined in a previous study (Nguyen, Huyen, et al., 2022).

### 2.6 Determination of the capacity to inhibit α‐amylase and α‐glucosidase activities

The inhibition of α‐amylase and α‐glucosidase activities was measured using the methods reported in a previous publication (Nguyen, Huyen, et al., 2022).

### 2.7 Maintenance of zebrafish

All animal procedures were conducted in accordance with the “Act on the Conservation and Sustainable Use of Biological Diversity via Regulations on the Use of Living Modified Organisms,” the “Cartagena Protocol on Biosafety,” and the “Guide for the Care and Use of Laboratory Animals at the Biotechnology Centre of Ho Chi Minh City.” All research was authorized by the Biotechnology Centre of Ho Chi Minh City in January 2013 with decision No.02/QD‐CNSH.

Zebrafish (*Danio rerio*) were supplied by Laboratory Animals at the Biotechnology Centre of Ho Chi Minh City and reared in standard conditions according to the laboratory using standards for zebrafish (Westerfield, 2000). The dechlorinated tap water was filtered through a filtration system and the Ultraviolet filter was used to minimize contaminants. The culture medium was kept at a pH of 6.8–7.0, a temperature of 28 ± 1°C, a light cycle of 14 h (light)/10 h (dark), and adequate oxygen was supplied by an air pump. The fish was placed at the rate of 15 adults in each 4 L tank (Westerfield, 2000).

### 2.8 Postprandial blood glucose level assay

Adult zebrafish (300–400 mg BW/fish) fasted for 12 h before the experiment. The fish after the fasting period was put in freezing water at 4°C to induce hypothermia. The anesthetic stage was detected when the fish lost balance, and operculum movements without response. The anesthetic fishes were then force‐fed following the standard method with slight modifications (Collymore et al., 2013). The fishes were divided into six groups (10 fishes/group) and treated with maltodextrin (MD), acarbose, *SZL* extract, and its fractions, as described below:

Group 1: Maltodextrin was administered at 1 mg/g BW (negative control).
Group 2: Maltodextrin (1 mg/g BW) + EAF (75 mg/kg BW).
Group 3: Maltodextrin (1 mg/g BW) + BF (75 mg/kg BW).
Group 4: Maltodextrin (1 mg/g BW) + WF (75 mg/kg BW).
Group 5: Maltodextrin (1 mg/g BW) + CE (75 mg/kg BW).
Group 6: Maltodextrin (1 mg/g BW) + Acarbose (75 mg/kg BW; positive control).

After feeding for 30, 60, 120, and 180 min, the blood was collected by the standard method (Zang et al., 2015). Postprandial blood glucose level was measured using a glucometer (Multicare‐In, Biochemical Systems International).

### 2.9 Ultra‐performance liquid chromatography (UPLC) analysis

The phenolic constituents in the extract and its fractions were figured out using the Thermo‐Ultimate 3000UPLC system (Thermo Scientific) with the gradient method as described by Nguyen, Huyen, et al. (2022).

### 2.10 Data analysis

The mean values of each treatment were compared using an ANOVA test followed by a post hoc test (STATGRAPHICS Centurion XV). The LSD test identified significant variations in means (*p* < .05). The results were reported as the mean ± standard deviation (SD). The PCA was performed using R software version 4.1.2 (R Core Team, 2021) with the package FactoMineR (Lê et al., 2008).

## 3 RESULTS AND DISCUSSION

### 3.1 The total polyphenol and flavonoid contents

Polyphenol and flavonoids were found to be a rich source of *Syzygium* sp. which exhibits several valuable bioactivities such as anti‐inflammatory, cardiometabolic, antioxidant, and antidiabetic activities (Adefegha & Oboh, 2012; Chagas et al., 2018; Gajera et al., 2017; Priya et al., 2017; Thomas et al., 2022). In our current work, the n‐hexane and chloroform fractions were collected in extremely insignificant amounts that were not enough quantity for further characterization and bioactivity tests. As an observation, *SZL* extract contains high quantities of polyphenols and flavonoids. The total polyphenol and flavonoid contents of CE were 531.64 mg GAE/g and 473.44 mg QE/g dry weight (DW), respectively (Table 1). These results were in good agreement with previous studies on *Syzygium* sp. (Priya et al., 2017; Ruan et al., 2008). According to Mai et al. (2007), the total polyphenol content in *SZL* leaf is the highest among 11 species of culinary plants that grow naturally in Vietnam, with values of 176.9 mg/g DW and 251.7 mg/g DW for crude and methanolic extract, respectively. Among all fractions, the EAF had the highest total polyphenol and flavonoid contents, followed by the BF, while the WF had the lowest contents. In comparison with WF, the EAF and BF showed a superior performance to extract polyphenols and flavonoids from the CE (Table 1). These results showed that the polyphenols and flavonoids in the trunk‐bark of *SZL* have similar polarity to ethyl acetate and n‐butanol, allowing them to be fractionated readily using these solvents.

**TABLE 1:** Total polyphenol, flavonoid contents, antioxidant activity, starch‐hydrolyzing enzyme inhibitory activity of *SZL* extract and its fractions.

Extracts/control	TPC (mg GAE/g DW)	TFC (mg QE/g DW)	Antioxidant activity (g TE/g DW)		Enzyme inhibition IC_50_ (μg/mL)	
			DPPH	ABTS	α‐Amylase	α‐Glucosidase
CE	531.64 ± 5.47^c^	473.44 ± 37.08^c^	1.26 ± 0.01^b^	1.40 ± 0.06^d^	4.19 ± 0.01^d^	0.41 ± 0.02^c^
EAF	904.11 ± 3.39^a^	897.66 ± 23.34^a^	1.50 ± 0.08^a^	3.27 ± 0.09^a^	12.32 ± 0.01^a^	0.43 ± 0.02^c^
BF	810.90 ± 11.53^b^	710.21 ± 1.40^b^	1.12 ± 0.05^c^	1.51 ± 0.06^b^	5.88 ± 0.01^b^	0.37 ± 0.01^d^
WF	482.42 ± 9.79^d^	427.30 ± 8.87^d^	1.05 ± 0.01^d^	1.70 ± 0.13^c^	4.52 ± 0.01^c^	0.69 ± 0.02^b^
Acarbose	—	—	—	—	5.91 ± 0.13^b^	16,037.86 ± 5.08^a^

*Note*: Results expressed mean ± SD of multiple measurements. Different labels (a–d) indicate a significant difference within the column at (*p* < .05).

Abbreviations: “—”, not detected; BF, n‐butanol fraction; CE, crude extract; DW, dry weight of extract/fraction; EF, ethyl acetate fraction; GAE, gallic acid equivalent; QE, quercetin equivalent; TE, Trolox equivalent; TF, total flavonoid content; TPC, total polyphenol content; WF, water fraction.

### 3.2 DPPH ^•^ radical scavenging activity

The DPPH^•^ radical scavenging activity results were expressed in gram Trolox equivalent (TE) per gram DW of extract/fraction which was observed to be significantly different amongst extract and fractions (*p* < .05; Table 1). The maximum capacity was observed in the EAF, whereas the lowest was observed in the WF. Indeed, EAF demonstrated the highest DPPH^•^ radical scavenging activity of 1.5 g TE/g DW followed by CE, BF, and WF of 1.26, 1.12, and 1.05 g TE/g DW, respectively. These findings indicated that the strongest DPPH^•^ radical scavenging of EAF could be due to high contents of moderately polar molecules, polyphenol, and flavonoid levels that have high antioxidative activities. These results were consistent with the previous studies on *S. cumini* according to Alawiyah and Senania (2021) and Ruan et al. (2008) reported. Interestingly, while having higher polyphenol and flavonoid contents, the DPPH^•^ radical scavenging capability of BF was lower than that of CE. The reason is possible that the major components of BF do not possess DPPH^•^ radical scavenging activity. Thus, an additional investigation of the chemical components of the *SZL* extract and its fractions was required.

### 3.3 ABTS ^•+^ radical scavenging activity

Like DPPH^•^ radical scavenging activity, EAF was determined to be relatively high in the ABTS^•+^ radical scavenging activity compared to all other fractions. The activity of this fraction was found to be greatest at 3.27 g TE/g DW, while the BF and WF showed a value of 1.4 g TE/g DW and 1.7 g TE/g DW, respectively (Table 1). Especially, all extracts and fractions exhibited greater ABTS^•+^ radical scavenging activity than that DPPH^•^ radical scavenging activity. The findings demonstrate that the EAF obtained most of the antioxidant compounds, the electron transfer compounds being the most abundant. In addition, the WF showed stronger ABTS^•+^ radical scavenging activity than that of BF and CE, suggesting that highly polar compounds have significantly higher antioxidative potential than nonpolar compounds. In literature, oxidative stress and inflammation engage in the onset and evolution of diabetes, as well as their repercussions (Kanwugu et al., 2022). Previous research highlighted the correlation between the antioxidant, anti‐inflammation, and antidiabetic potentials of *Syzygium* sp. (Arumugam et al., 2014). Thus, further investigation should be conducted to figure out the correlation between antioxidant and antidiabetic *SZL* bioactive components.

### 3.4 Potential for inhibition of starch‐hydrolyzing enzymes

#### 3.4.1 α‐Amylase inhibitory activity

The IC_50_ value of α‐amylase inhibitory activity revealed a significant difference between the extract to the fractions (Table 1). CE inhibited α‐amylase activity at the highest level, followed by WF (IC_50_: 4.19 and 4.52 μg/mL, respectively), and both had higher activity than BF and EAF. BF and acarbose exhibited no significant difference in activity (*p* > .05). Interestingly, EAF had the lowest activity despite having the greatest TPC, TFC, and antioxidant activity levels.

The α‐amylase inhibitory activity of *SZL* extract and its fractions was markedly higher as compared to *S. cumini* leaves (IC_50_: 39.9 μg/mL; Poongunran et al., 2017) or *S. aqueum* leaf extract (IC_50_: 8 μg/mL; Manaharan et al., 2012). These findings suggested that *SZL* extract is a reliable source of α‐amylase inhibitors, which would be useful in the treatment of type 2 diabetes. Several studies revealed a positive correlation between polyphenols content and α‐amylase inhibitory ability (Abdin et al., 2019; Adefegha & Oboh, 2012; Kim et al., 2020); however, in few cases, no correlation existed (Adisakwattana et al., 2011). These findings in this study suggested that the fractionated procedure may alter the component levels of α‐amylase inhibitors from fraction to fraction and have an influence on the α‐amylase inhibitory activity of extract and its fractions. Further research into the isolation and characterization of α‐amylase inhibitors is highly recommended.

#### 3.4.2 α‐Glucosidase inhibitory activity

The efficiency of α‐glucosidase inhibition, as represented by IC_50_ values, was shown to differ considerably between extract and fractions (Table 1). BF possessed the highest α‐glucosidase inhibitory activity with the IC_50_ value of 0.37 μg/mL, while there was no significant difference between EAF and CE with the IC_50_ values of 0.43 and 0.41 μg/mL, respectively. The WF showed the lowest activity with the IC_50_ value of 0.69 μg/mL (Table 1). These results are comparable to previous studies, the leaf extract of *SZL* showed the highest α‐glucosidase inhibition among four different promising plants with the IC_50_ of 0.1 mg/mL (Mai et al., 2007). The methanol *SZL* extract was found to have the highest α‐glucosidase inhibitory activity, with the IC_50_ value of 109 μg/mL (Nguyen et al., 2019). The *SZL* extract and its fraction could be an excellent source of α‐glucosidase inhibition activity as associated with the selection of an appropriate solvent. The most effective solvent and extraction processes for the active ingredients associated with α‐glucosidase inhibitors should be explored further.

According to the literature, an effective therapeutic strategy for treating diabetes by inhibiting starch‐hydrolyzing enzymes, such as α‐amylase and α‐glucosidase. When an inhibitor derived from medicinal plants was treated, it may reduce negative consequences by reducing aberrant fermentation or breakdown of polysaccharides in the colon due to α‐amylase activity (Wang et al., 2012). Our findings in this study revealed that the EAF and BF of *SZL* extracts could be an alternative treatment method for type 2 diabetes due to their substantial inhibition of α‐glucosidase and moderate inhibition of α‐amylase.

### 3.5 Postprandial antihyperglycemic activity on zebrafish model

**FIGURE 1:**
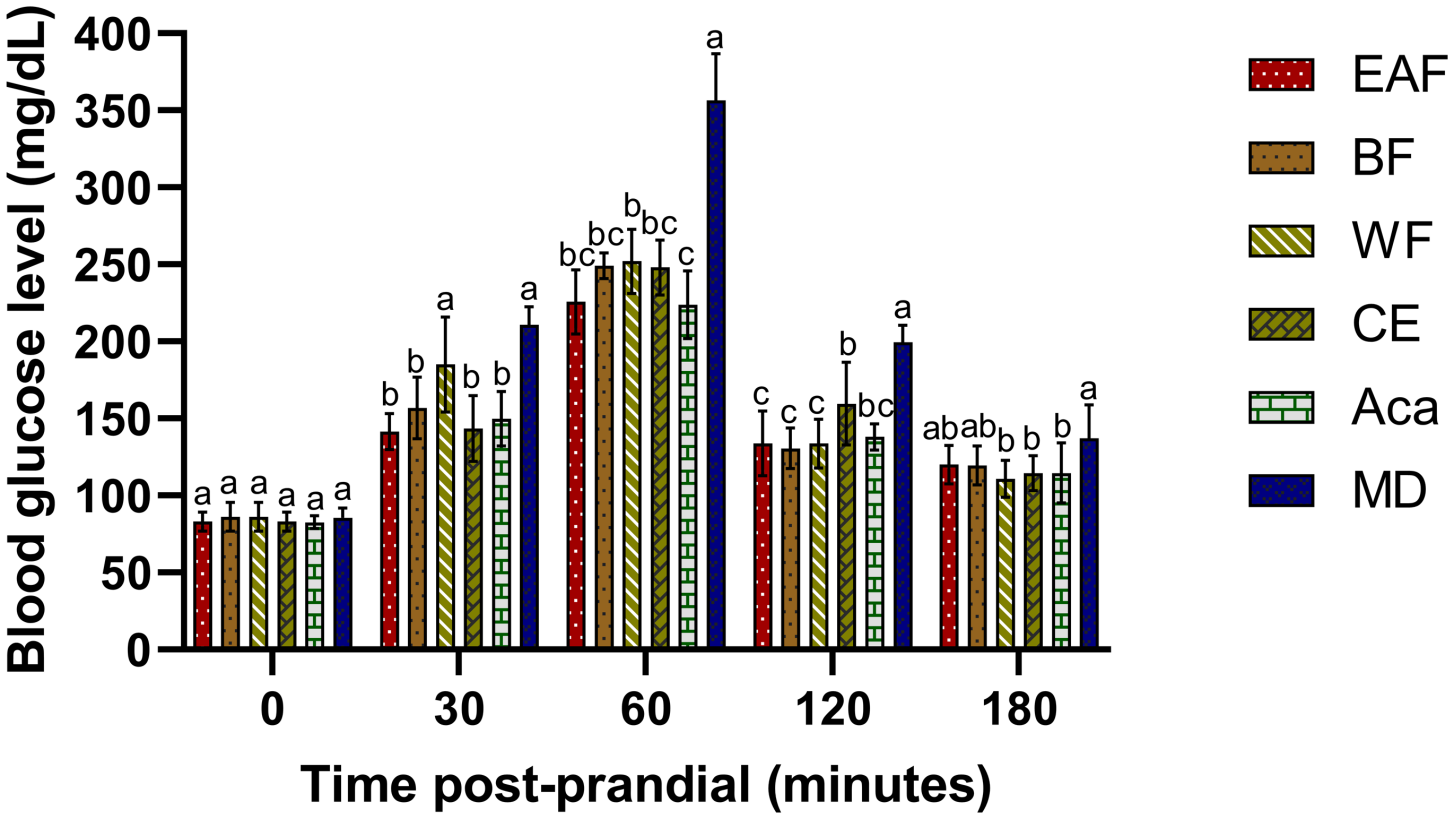
The effect of *SZL* extract, its fraction, and acarbose on blood glucose levels after oral maltodextrin loading in Zebrafish. *SZL* extract and its fraction (75 mg/kg BW), acarbose (75 mg/kg BW), and maltodextrin (1 mg/g BW) were given to zebrafish (*n* = 10) orally. Blood was drawn at 0, 30, 60, 120, and 180 min after feeding. Different labels (a–c) indicate a significant difference within the group of times (*p* < .05), Aca, acarbose; BF, n‐butanol fraction; CE, crude extract; EAF, ethyl acetate fraction; MD, maltodextrin; WF, water fraction.

Figure 1 depicts the postprandial blood glucose levels (PBGL) of zebrafish treated with *SZL* extract and its fractions. At the dose of 75 mg/kg body weight (BW), all the treatments of *SZL* extract and its fractions significantly lowered PBGL from 12% to 37% as compared to maltodextrin (MD) after feeding 30, 60, 120, and 180 min (*p* < .05). The PBGL of all zebrafish groups reached the highest peak after 60 min of feeding, and the strongest hypoglycemic activity of *SZL* extract and its fractions were also observed at this time. Specifically, the EAF, CE, BF, and WF treatments reduced PBGL by 29%–37% compared to the MD treatment, with PBGL values of 225.8, 248, 249.2, and 252 mg/dL, respectively, which were the same as the value of acarbose treatment (223.8 mg/dL). The positive effect remained after 180 min of feeding. *SZL* extract and its fractions treatments still showed a significant reduction in PBGL level from 13% (EAF, 120 mg/dL) to 19% (WF, 110.8 mg/dL) compared to MD treatment (137.2 mg/dL; *p* < .05). Especially, the hypoglycemic activity may differ from fraction to fraction over time. Indeed, EAF, BF, and CE exhibited better effects than WF until 120 min while WF showed better after 180 min. These findings revealed that the mechanism of action may be distinct and that the fractionation procedure altered the quantities of bioactive components, influencing plasma blood glucose levels over time.

In the previous reports, we found that methanolic *SZL* extract had significant hypoglycemic activity in mice at a dosage ranging from 100 to 200 mg/kg BW (Nguyen et al., 2019). In this work, we firstly reported the postprandial glycemic reduction of *SZL* extract and its fractions in a zebrafish model with a lower dosage and longer lasting activity.

### 3.6 UPLC analysis and phenolic compound properties

The most prevalent secondary metabolites found in plants are phenolic compounds which supplied a wide variety of bioactivity in general (Zhang et al., 2022). Gallic acid, catechin, epicatechin, caffeine, ethyl gallate, rutin, ellagic acid, quercetin, quercitrin, apigenin, and chlorogenic acid were among 11 recognized phenolic compounds discovered in the *SZL* extract and its fractions. Their concentration altered from the extract to the fractions (Table 2). Catechin was discovered to be the most abundant in CE, with the highest content (11,079.7 μg/g), followed by chlorogenic acid, rutin, ellagic acid, and epicatechin (5405.5–8714.81 μg/g), whereas gallic acid, ethyl gallate, quercetin, and quercitrin were discovered to be less abundant (2708.73–3514.38 μg/g). Caffeine and apigenin were found in extremely low amounts (447.13–493.46 μg/g; Table 2).

**TABLE 2:** The contents of the phenolic compounds in *SZL* extract and its fractions.

Phenolic compounds (μg/g DW)	Extract/fractions			
	CE	EAF	BF	WF
GA	2708.73 ± 0.02^b^	47,476.10 ± 0.24^a^	2296.90 ± 0.56^c^	560.627 ± 0.52^d^
Cat	11,079.70 ± 0.01^d^	70,040.80 ± 0.42^a^	16,876.90 ± 0.19^b^	15,454 ± 0.33^c^
E‐Cat	5405.4 ± 0.02^d^	43,851.00 ± 0.11^a^	7753.74 ± 0.41^b^	5879.54 ± 1.1^c^
Caf	493.46 ± 00^d^	4922.62 ± 0.41^a^	1257.84 ± 0.08^b^	717.77 ± 0.48^c^
EG	2657.29 ± 00^c^	5215.96 ± 0.29^a^	4167.87 ± 0.19^b^	1866.99 ± 0.33^d^
RT	7230.78 ± 0.01^c^	14,301.30 ± 0.72^a^	13,714.90 ± 0.40^b^	3454.38 ± 0.68^d^
EA	5603.90 ± 0.01^b^	23,183.90 ± 0.26^a^	2344.92 ± 0.40^c^	761.42 ± 0.63^d^
Querce	2460.51 ± 0.02^b^	3954.62 ± 0.42^a^	1048.00 ± 0.27^c^	604.5 ± 0.84^d^
Ap	447.13 ± 00^b^	1345.67 ± 0.47^a^	—	—
ChA	8714.81 ± 0.01^c^	21,945.10 ± 0.24^a^	9264.81 ± 0.26^b^	4598.40 ± 0.45^d^
Querci	3514.38 ± 0.04^c^	25,237.70 ± 0.48^a^	10,845.7 ± 0.69^b^	2630.44 ± 0.78^d^

*Note*: Results expressed mean ± SD of multiple measurements. Different labels (a–d) indicate a significant difference within the row (*p* < .05).

Abbreviations: Ap, apigenin; BF, n‐butanol fraction; Caf, caffein; Cat, catechin; CE, crude extract; ChA, chlorogenic acid; DW, dry weight of extract/fraction; EA, ellagic acid; EAF, ethyl acetate fraction; E‐Cat, epicatechin; EG, ethyl gallate; GA, gallic acid; Querce, quercetin; Querci, quercitrin; RT, rutin; WF, water fraction.

Each phenolic compound was extracted in varying concentrations through fractionation by different solvents. All phenolic compounds were found to be the highest amount in EAF when compared with BF and WF (Table 2). Gallic acid was the most abundant in EAF (17.5 folds of CE), followed by caffeine, epicatechin, quercitrin, and catechin (9.98, 8.1, 7.2, 6.3 folds of CE, respectively), and other compounds ranging from 1.9 to 3.0 folds of CE. Whereas seven compounds including quercitrin, caffeine, rutin, ethyl gallate, catechin, epicatechin, and chlorogenic acid were concentrated in BF, with levels ranging from 1.06 (chlorogenic acid) to 3.1 folds (quercitrin) of CE, and only three compounds including caffeine, catechin, and epicatechin were found to be increasing level in WF (1.45, 1.12, and 1.1 folds of CE, respectively; Table 2). The concentration of individual phenolic compounds and their ratio in the *SZL* extract and its fractions may influence their bioactivities. However, the primary compound that has the greatest impact on bioactivities must be investigated further.

### 3.7 Correlation analysis and the principal component analysis

Pearson's correlations (*r*) between TPC, TFC, DPPH^•^, and ABTS^•+^ radical scavenging activities, and phenolic components along with α‐glucosidase and α‐amylase inhibitory activities were observed and arranged from lowest to highest (Table 3). The high correlation between TPC and TFC (*r* = .98, *p* < .001) revealed that flavonoids are the primary components of phenolic substances. The flavonoid components also exhibited stronger relationship to DPPH^•^ (*r* = .73, *p* < .001) and ABTS^•+^ (*r* = .78, *p* < .001) radical scavenging abilities compared to TPC (*r* = .62, and *r* = .67, *p* < .001, respectively). This finding supports prior research that found flavonoids to be the responsible components for the antioxidant activity of a medicinal plant extract and food (Ali et al., 2020; Manaharan et al., 2012; Wang et al., 2012; Zeb, 2020). Moreover, the phenolic components in *SZL* extract and its fractions play diverse roles in antioxidant activities. Indeed, the DPPH^•^ radical scavenging capacity showed high correlation (*p* < .001) with ellagic acid (*r* = .94), quercetin (*r* = .97), apigenin (*r* = .96), and chlorogenic acid (*r* = .93), while the ABTS^•+^ neutralizing ability exhibited greater correlation with gallic acid (*r* = .98), catechin (*r* = .98), epicatechin (*r* = .98), caffeine (*r* = .97), ellagic acid (*r* = .94), chlorogenic acid (*r* = .91), and quercitrin (*r* = .91; Table 3). These results suggested that the higher levels of ellagic acid, quercetin, and apigenin in CE may support their DPPH^•^ radical scavenging activity better than that of BF, and WF, which had lower levels of ellagic acid, quercetin, and absence of apigenin (Table 2). Similarly, the higher levels of catechin, epicatechin, and caffeine in WF may be responsible for its ABTS^•+^ radical scavenging activity better than that of CE and BF.

**TABLE 3:** Pearson's correlation matrix among total phenolic and flavonoid contents (TPC and TFC), DPPH^•^ and ABTS^•+^ scavenging (DPPH and ABTS) activities, α‐glucosidase, and α‐amylase inhibitory activities (a_AI & a_GI), phenolic compounds of SZL extract and its fractions.

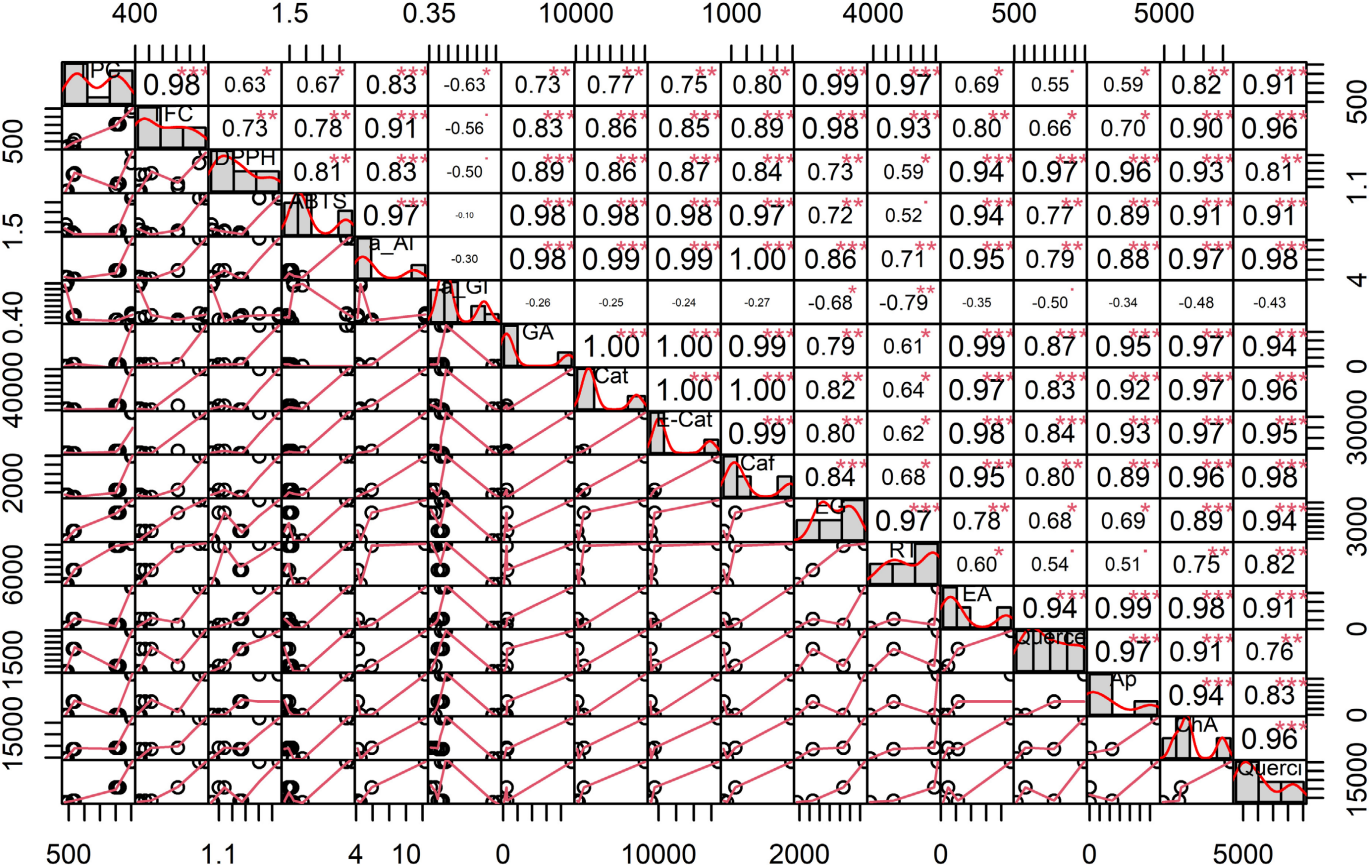

Interestingly, a strong positive correlation between phenolic compounds with the IC_50_ value of α‐amylase inhibitory activity was observed (Table 3). This situation implied that the increasing concentration of these compounds is suggested to reduce the α‐amylase inhibitory activity. In literature, the phenolic interaction with each other affects their α‐amylase inhibitory activity due to decreasing their interaction with enzymes (Sultana et al., 2020). A previous study reported that an elevated level of phenolic compounds in yellow raspberries did not increase α‐amylase inhibition (Grussu et al., 2011). Thus, these findings suggested the behavior of α‐amylase inhibitory activity of *SZL* extract and its fraction may affect by the interaction of specific types of phenolic compounds than their total concentration level.

In terms of α‐glucosidase inhibition, only ethyl gallate and rutin showed a significant association with *r* = −.68 (*p* < .016) and *r* = −.79 (*p* < .0025), respectively, whereas other compounds showed an insignificant correlation (Table 3). Furthermore, ethyl gallate and rutin may differ from other phenolic compounds in their activity. They have a strong association with each other (*r* = .97, *p* < .001), but only a moderate link with the other compounds (excluding quercitrin in the case of ethyl gallate; Table 3). This discovery demonstrated that ethyl gallate and rutin may become intertwined during fractionation and that changes in concentrated levels over fractions may impact the α‐glucosidase inhibitory activity.

**FIGURE 2:**
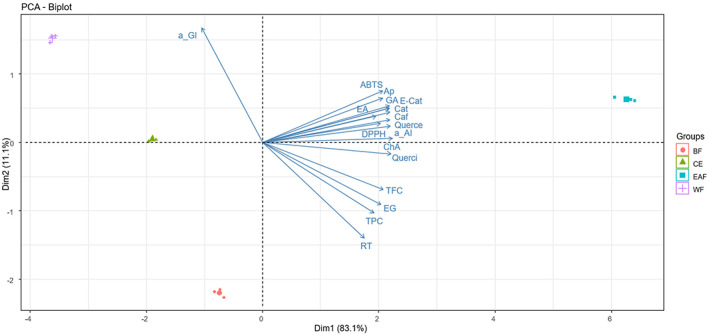
Principal Component Analysis (PCA) Biplot for total phenolic and flavonoid contents, antioxidant activity, enzyme inhibitory activity, phenolic compounds of SZL extract, and its fractions. CE, crude extract; EAF, ethyl acetate fraction; BF, n‐butanol fraction; WF, water fraction; GA, gallic acid; Cat, catechin; E‐Cat, epicatechin; Caf, caffein; EG, ethyl gallate; RT, rutin; EA, ellagic acid; Querce, quercetin; Querci, quercitrin; Ap, apigenin; ChA, chlorogenic acid; total polyphenol and flavonoid contents (TPC and TFC), DPPH^•^ and ABTS^•+^ scavenging (DPPH and ABTS) activities, α‐glucosidase, and α‐amylase inhibitory activities (a_AI & a_GI).

The PCA of *SZL* extract and fractions is shown in Figure 2. The two dimensions described an eigenvalue of about 93%, accounting for a variance of 83.1% by Dim. 1 and 11.1% by Dim. 2. Overall, different fractions demonstrated distinct separate situations with CE. Due to their different polarities, EAF was separated by Dim. 1 with WF, BF, and CE. On the other hand, BF was distinct from WF on opposite sites by Dim 2, and both were well separated with CE. These findings showed that the metabolite compositions of *SZL* extract and its fractions were completely different from each of them. These circumstances may have an impact on their bioactivities, which represent by the direction of antioxidant and enzyme inhibitory activities (Figure 2). In comparison with water, n‐butanol and ethyl acetate performed better as solvents for phenolic and flavonoid extractions from the CE. Among them, ethyl acetate has the best collection of components with high antioxidant activity. These abilities were demonstrated by the direction of DPPH^•^ and ABTS^•+^ scavenging activities on the positive side of Dim. 1 and Dim. 2, which is the side of EAF. Interestingly, gallic acid, catechin, epicatechin, ellagic acid, quercetin, caffeine, and apigenin had better contributions in antioxidants than that of chlorogenic acid, quercitrin, and especially ethyl gallate and rutin, which were separated in the opposite side of Dim. 2 (Figure 2). The direction of phenolic compounds in Dim. 2 also showed that the condensed level and interaction of gallic acid, catechin, epicatechin, ellagic acid, quercetin, and apigenin decreased the α‐amylase inhibitory activity of EAF stronger than that of ethyl gallate, rutin, quercitrin, and chlorogenic acid (Figure 2). The results could imply that the fractionation process had concentrated and changed the ratio of bioactive components, thereby affecting the α‐amylase inhibitory activity. In terms of α‐glucosidase inhibition, the opposite side direction of the IC_50_ value of α‐glucosidase inhibitory activity revealed the relative components, which may be gathered by the BF rather than the EAF. Indeed, rutin and ethyl gallate correlated with the IC_50_ value of α‐glucosidase inhibitory activity oppositely (Figure 2), and they correlated with BF in Dim. 2. These findings suggested that the greater amount of ethyl gallate and rutin in BF may increase α‐glucosidase inhibitory action in contrast to EAF, which had a lower ratio of them to others (Table 2).

## 4 CONCLUSION

The findings in this study show that the trunk‐bark of *SZL* is a rich source of phenolic compounds such as gallic acid, catechin, epicatechin, caffeine, ethyl gallate, rutin, ellagic acid, quercetin, quercitrin, apigenin, and chlorogenic acid with relatively high antioxidant and in vitro antidiabetes properties. The solvent used for fractionation affects the bioactivities of a fraction due to variations in the types and concentrations of phenolic compounds. EAF has the highest phenolic compounds concentration and antioxidant activity, while BF has the highest α‐glucosidase inhibitory activity and CE has the highest α‐amylase inhibitory activity. The condensed level and interaction of gallic acid, catechin, epicatechin, ellagic acid, quercetin, and apigenin decreased the α‐amylase inhibition activity while the greater amount of ethyl gallate and rutin increased α‐glucosidase inhibitory activity. Moreover, *SZL* extract and its fractions were found to be potent antihyperglycemic agents in zebrafish model possibilities. Further clinical research is needed to confirm the antidiabetic effect of major bioactive components of the *SZL* extract and its fractions.

## AUTHOR CONTRIBUTIONS

**Minh‐Trung Nguyen:** Data curation (equal); investigation (equal); methodology (equal); writing – original draft (lead). **Bich Huyen Bui Thi:** Investigation (equal); methodology (equal). **Shila Maskey:** Writing – review and editing (supporting). **Minh‐Dinh Tran:** Investigation (equal); methodology (equal). **Quang‐Vinh Nguyen:** Conceptualization (lead); data curation (lead); investigation (lead); methodology (lead); writing – review and editing (lead).

## FUNDING INFORMATION

The research leading to these results received funding from The National Foundation for Science and Technology Development (NAFOSTED) under Grant Agreement No 106.99‐2020.17.

## CONFLICT OF INTEREST STATEMENT

The authors declare that they have no known competing financial interests or personal relationships that could have appeared to influence the work reported in this paper.

## ETHICAL APPROVAL

All animal procedures were carried out in accordance with the “Act on the Conservation and Sustainable Use of Biological Diversity via Regulations on the Use of Living Modified Organisms,” the “Cartagena Protocol on Biosafety,” and the “Guide for the Care and Use of Laboratory Animals at the Biotechnology Centre of Ho Chi Minh City.” All research was authorized by the Biotechnology Centre of Ho Chi Minh City in January 2013 with decision No.02/QD‐CNSH.

## Data Availability

The data that support the findings of this study are available from the corresponding author upon reasonable request.

## References

[fsn33373-bib-0001] Abdin, M. , Hamed, Y. S. , Akhtar, H. M. S. , Chen, D. , Mukhtar, S. , Wan, P. , Riaz, A. , & Zeng, X. (2019). Extraction optimization, antioxidant activity, and inhibition on α‐amylase and pancreatic lipase of polyphenols from the seeds of *Syzygium cumini* . International Journal of Food Science & Technology, 54(6), 2084–2093. 10.1111/ijfs.14112

[fsn33373-bib-0002] Adefegha, S. A. , & Oboh, G. (2012). In vitro inhibition activity of polyphenol‐rich extracts from *Syzygium aromaticum* (L.) Merr. & Perry (clove) buds against carbohydrate hydrolyzing enzymes linked to type 2 diabetes and Fe^2+^‐induced lipid peroxidation in rat pancreas. Asian Pacific Journal of Tropical Biomedicine, 2(10), 774–781. 10.1016/S2221-1691(12)60228-7 23569846 PMC3609220

[fsn33373-bib-0003] Adisakwattana, S. , Lerdsuwankij, O. , Poputtachai, U. , Minipun, A. , & Suparpprom, C. (2011). Inhibitory activity of cinnamon bark species and their combination effect with acarbose against intestinal α‐glucosidase and pancreatic α‐amylase. Plant Foods for Human Nutrition, 66(2), 143–148. 10.1007/s11130-011-0226-4 21538147

[fsn33373-bib-0004] Alawiyah, A. L. , & Senania, A. (2021). Antioxidant activity and bioactive compounds of ethyl acetate fractions from *Syzygium cumini* wood stem. ALKIMIA: Jurnal Ilmu Kimia dan Terapan, 5(1), 93–101.

[fsn33373-bib-0005] Ali, S. S. , Ahsan, H. , Zia, M. K. , Siddiqui, T. , & Khan, F. H. (2020). Understanding oxidants and antioxidants: Classical team with new players. Journal of Food Biochemistry, 44, e13145. 10.1111/jfbc.13145 31960481

[fsn33373-bib-0006] Arumugam, B. , Manaharan, T. , Heng, C. K. , Kuppusamy, U. R. , & Palanisamy, U. D. (2014). Antioxidant and antiglycemic potentials of a standardized extract of *Syzygium malaccense* . LWT‐ Food Science and Technology, 59(2), 707–712. 10.1016/j.lwt.2014.06.041

[fsn33373-bib-0007] Bashary, R. , Vyas, M. , Nayak, S. K. , Suttee, A. , Verma, S. , Narang, R. , & Khatik, G. L. (2020). An insight of alpha‐amylase inhibitors as a valuable tool in the management of type 2 diabetes mellitus. Current Diabetes Reviews, 16(2), 117–136. 10.2174/1573399815666190618093315 31237215

[fsn33373-bib-0008] Bindu, J. , & Narendhirakannan, R. T. (2019). Role of medicinal plants in the management of diabetes mellitus: A review. Biotech, 9(1), 4. 10.1007/s13205-018-1528-0 PMC6291410 30555770

[fsn33373-bib-0009] Chagas, V. T. , Coelho, R. , Gaspar, R. S. , da Silva, S. A. , Mastrogiovanni, M. , Mendonça, C. J. , Ribeiro, M. , Paes, A. , & Trostchansky, A. (2018). Protective effects of a polyphenol‐rich extract from *Syzygium cumini* (L.) Skeels leaf on oxidative stress‐induced diabetic rats. Oxidative Medicine and Cellular Longevity, 2018, 5386079. 10.1155/2018/5386079 30046378 PMC6038589

[fsn33373-bib-0010] Collymore, C. , Rasmussen, S. , & Tolwani, R. J. (2013). Gavaging adult zebrafish. Journal of Visualized Experiments, (78), e50691. 10.3791/50691 PMC3855001 23962977

[fsn33373-bib-0011] Dirir, A. M. , Daou, M. , Yousef, A. F. , & Yousef, L. F. (2021). A review of alpha‐glucosidase inhibitors from plants as potential candidates for the treatment of type‐2 diabetes. Phytochemistry Reviews, 1–31, 1049–1079. 10.1007/s11101-021-09773-1 PMC8364835 34421444

[fsn33373-bib-0012] Franco, R. R. , Ribeiro Zabisky, L. F. , de Lima, P. , Junior, J. , Mota Alves, V. H. , Justino, A. B. , Saraiva, A. L. , Goulart, L. R. , & Espindola, F. S. (2020). Antidiabetic effects of *Syzygium cumini* leaves: A non‐hemolytic plant with potential against process of oxidation, glycation, inflammation and digestive enzymes catalysis. Journal of Ethnopharmacology, 261, 113132. 10.1016/j.jep.2020.113132 32673709

[fsn33373-bib-0013] Gajera, H. P. , Gevariya, S. N. , Hirpara, D. G. , Patel, S. V. , & Golakiya, B. A. (2017). Antidiabetic and antioxidant functionality associated with phenolic constituents from fruit parts of indigenous black jamun (*Syzygium cumini* L.) landraces. Journal of Food Science and Technology, 54(10), 3180–3191. 10.1007/s13197-017-2756-8 28974803 PMC5602981

[fsn33373-bib-0014] Grussu, D. , Stewart, D. , & McDougall, G. J. (2011). Berry polyphenols inhibit α‐amylase in vitro: Identifying active components in rowanberry and raspberry. Journal of Agricultural and Food Chemistry, 59(6), 2324–2331. 10.1021/jf1045359 21329358

[fsn33373-bib-0015] Kanwugu, O. N. , Glukhareva, T. V. , Danilova, I. G. , & Kovaleva, E. G. (2022). Natural antioxidants in diabetes treatment and management: Prospects of astaxanthin. Critical Reviews in Food Science and Nutrition, 62(18), 5005–5028. 10.1080/10408398.2021.1881434 33591215

[fsn33373-bib-0016] Kim, S. , Semple, S. J. , Simpson, B. S. , & Deo, P. (2020). Antioxidant and antiglycation activities of *Syzygium paniculatum* Gaertn and inhibition of digestive enzymes relevant to type 2 diabetes mellitus. Plant Foods for Human Nutrition, 75(4), 621–627. 10.1007/s11130-020-00858-4 33009631

[fsn33373-bib-0017] Lê, S. , Josse, J. , & Husson, F. (2008). FactoMineR: An R package for multivariate analysis. Journal of Statistical Software, 25, 1–18. 10.18637/jss.v025.i01

[fsn33373-bib-0019] Mai, T. T. , Thu, N. N. , Tien, P. G. , & Van Chuyen, N. (2007). Alpha‐glucosidase inhibitory and antioxidant activities of Vietnamese edible plants and their relationships with polyphenol contents. Journal of Nutritional Science and Vitaminology (Tokyo), 53(3), 267–276. 10.3177/jnsv.53.267 17874833

[fsn33373-bib-0020] Manaharan, T. , Appleton, D. , Cheng, H. M. , & Palanisamy, U. D. (2012). Flavonoids isolated from *Syzygium aqueum* leaf extract as potential antihyperglycaemic agents. Food Chemistry, 132(4), 1802–1807. 10.1016/j.foodchem.2011.11.147

[fsn33373-bib-0021] Nguyen, M. T. , Van Chuyen, H. , Tran, M. D. , & Nguyen, Q. V. (2022). Microencapsulation of *Syzygium zeylanicum* (L.) DC. Extract using spray drying: Effects of wall materials on physicochemical characteristics and biological activities of the microcapsules. Journal of Food Processing and Preservation, 46(7), e16647. 10.1111/jfpp.16647

[fsn33373-bib-0022] Nguyen, Q. V. , Eun, J. B. , Wang, S. L. , Nguyen, D. H. , Tran, T. N. , & Nguyen, A. D. (2016). Anti‐oxidant and antidiabetic effects of some medicinal plants belong to *Terminalia* species collected in Dak Lak Province, Vietnam. Research on Chemical Intermediates, 42(6), 5859–5871. 10.1007/s11164-015-2409-3

[fsn33373-bib-0023] Nguyen, Q. V. , Huyen, B. , Thi, B. , Tran, M. D. , Nguyen, M. T. , Doan, M. D. , Nguyen, A. D. , Minh Le, T. , Tran, V. C. , & Pham, T. N. (2022). Impact of different drying temperatures on In vitro antioxidant and antidiabetic activities and phenolic compounds of Wild guava leaves collected in the Central Highland of Vietnam. Natural Product Communications, 17, 1934578X2210953. 10.1177/1934578X221095349

[fsn33373-bib-0024] Nguyen Quang, V. , Hung, P. V. , & Nguyen, A. D. (2021). Antioxidant and hypoglycemic activities of various solvent fractions of methanol extract of *Terminalia alata* Heyne ex Roth trunk‐bark. Nova Biotechnologica et Chimica, 20(1), 748. 10.36547/nbc.748

[fsn33373-bib-0025] Nguyen, V. B. , Wang, S. L. , Nguyen, T. H. , Doan, C. T. , Tran, T. N. , Kuo, Y. H. , Nguyen, Q. V. , & Nguyen, A. D. (2019). New indications of potential rat intestinal α‐glucosidase inhibition by *Syzygium zeylanicum* (L.) and its hypoglycemic effect in mice. Research on Chemical Intermediates, 45(12), 6061–6071. 10.1007/s11164-019-04019-4

[fsn33373-bib-0027] Poongunran, J. , Perera, H. K. , Jayasinghe, L. , Fernando, I. T. , Sivakanesan, R. , Araya, H. , & Fujimoto, Y. (2017). Bioassay‐guided fractionation and identification of alpha‐amylase inhibitors from *Syzygium cumini* leaves. Pharmaceutical Biology, 55(1), 206–211. 10.1080/13880209.2016.1257031 27927056 PMC6130705

[fsn33373-bib-0028] Priya, S. H. , Prakasan, N. , & Purushothaman, J. (2017). Antioxidant activity, phenolic‐flavonoid content and high‐performance liquid chromatography profiling of three different variants of *Syzygium cumini* seeds: A comparative study. Journal of Intercultural Ethnopharmacology, 6(1), 107–114. 10.5455/jice.20161229055555 28163968 PMC5289079

[fsn33373-bib-0029] R Core Team . (2021). R: A language and environment for statistical computing. R Foundation for Statistical Computing. https://www.R‐project.org/

[fsn33373-bib-0030] Ruan, Z. P. , Zhang, L. L. , & Lin, Y. M. (2008). Evaluation of the antioxidant activity of *Syzygium cumini* leaves. Molecules, 13(10), 2545–2556. 10.3390/molecules13102545 18927517 PMC6245362

[fsn33373-bib-0031] Shori, A. B. (2015). Screening of antidiabetic and antioxidant activities of medicinal plants. Journal of Integrative Medicine, 13(5), 297–305. 10.1016/S2095-4964(15)60193-5 26343100

[fsn33373-bib-0032] Sultana, R. , Alashi, A. M. , Islam, K. , Saifullah, M. , Haque, C. E. , & Aluko, R. E. (2020). Inhibitory activities of polyphenolic extracts of Bangladeshi vegetables against alpha‐amylase, alpha‐glucosidase, pancreatic lipase, renin, and angiotensin‐converting enzyme. Food, 9(7), 844. 10.3390/foods9070844 PMC7404479 32610462

[fsn33373-bib-0033] Sun, H. , Saeedi, P. , Karuranga, S. , Pinkepank, M. , Ogurtsova, K. , Duncan, B. B. , Stein, C. , Basit, A. , Chan, J. C. N. , Mbanya, J. C. , Pavkov, M. E. , Ramachandaran, A. , Wild, S. H. , James, S. , Herman, W. H. , Zhang, P. , Bommer, C. , Kuo, S. , Boyko, E. J. , & Magliano, D. J. (2022). IDF diabetes atlas: Global, regional and country‐level diabetes prevalence estimates for 2021 and projections for 2045. Diabetes Research and Clinical Practice, 183, 109–119. 10.1016/j.diabres.2021.109119 PMC11057359 34879977

[fsn33373-bib-0034] Thomas, J. , Patel, A. , das Sivadasan, S. , Sreevallabhan, S. , Illathu Madhavamenon, K. , & Mohanan, R. (2022). Clove bud (*Syzygium aromaticum* L.) polyphenol helps to mitigate metabolic syndrome by establishing intracellular redox homeostasis and glucose metabolism: A randomized, double‐blinded, active‐controlled comparative study. Journal of Functional Foods, 98, 105273. 10.1016/j.jff.2022.105273

[fsn33373-bib-0035] Tiwari, P. (2015). Recent trends in therapeutic approaches for diabetes management: A comprehensive update. Journal of Diabetes Research, 2015, 340838. 10.1155/2015/340838 26273667 PMC4530263

[fsn33373-bib-0036] Wang, Y. , Huang, S. , Shao, S. , Qian, L. , & Xu, P. (2012). Studies on bioactivities of tea (*Camellia sinensis* L.) fruit peel extracts: Antioxidant activity and inhibitory potential against α‐glucosidase and α‐amylase in vitro. Industrial Crops and Products, 37(1), 520–526. 10.1016/j.indcrop.2011.07.031

[fsn33373-bib-0037] Westerfield, M. (2000). The zebrafish book. A guide for the laboratory use of zebrafish (Danio rerio) (4th ed.). University of Oregon Press. http://zfin.org/zf_info/zfbook/zfbk.html

[fsn33373-bib-0038] Willcox, M. L. , Elugbaju, C. , Al‐Anbaki, M. , Lown, M. , & Graz, B. (2021). Effectiveness of medicinal plants for Glycaemic control in type 2 diabetes: An overview of meta‐analyses of clinical trials [systematic review]. Frontiers in Pharmacology, 12, 777561. 10.3389/fphar.2021.777561 34899340 PMC8662558

[fsn33373-bib-0039] Yadav, S. S. , Meshram, G. A. , Shinde, D. , Patil, R. C. , Manohar, S. M. , & Upadhye, M. V. (2011). Antibacterial and anticancer activity of bioactive fraction of *Syzygium cumini* L. seeds. HAYATI Journal of Biosciences, 18(3), 118–122. 10.4308/hjb.18.3.118

[fsn33373-bib-0040] Zang, L. , Shimada, Y. , Nishimura, Y. , Tanaka, T. , & Nishimura, N. (2015). Repeated blood collection for blood tests in adult zebrafish. Journal of Visualized Experiments, (102), e53272. 10.3791/53272 26383512 PMC4692578

[fsn33373-bib-0041] Zeb, A. (2020). Concept, mechanism, and applications of phenolic antioxidants in foods. Journal of Food Biochemistry, 44, e13394. 10.1111/jfbc.13394 32691460

[fsn33373-bib-0042] Zhang, Y. , Cai, P. , Cheng, G. , & Zhang, Y. (2022). A brief review of phenolic compounds identified from plants: Their extraction, analysis, and biological activity. Natural Product Communications, 17(1), 1934578X211069721. 10.1177/1934578X211069721

